# Analysis of Vascular Mechanical Characteristics after Coronary Degradable Stent Implantation

**DOI:** 10.1155/2019/8265374

**Published:** 2019-11-20

**Authors:** Hao Ding, Ying Zhang, Yujia Liu, Chunxun Shi, Zhichao Nie, Haoyu Liu, Yuling Gu

**Affiliations:** ^1^School of Medical Instrument, Shanghai University of Medicine & Health Sciences, Shanghai 201318, China; ^2^School of Medical Instrument and Food Engineering, University of Shanghai for Science and Technology, Shanghai 200093, China; ^3^Research and Development Department, Shanghai Naturethink Life Science & Technology Co., Ltd., Shanghai 201809, China

## Abstract

**Purpose:**

To explore the effect of vascular stress changes on endothelial function recovery and vascular restenosis inhibition, under the condition of dynamic degradation process of the degradable stent.

**Methods:**

Fitting the material parameters of the hyperelastic vascular constitutive relationship, the stress distribution of the intima of the blood vessel before the stent was implanted and during the dynamic degradation was calculated by numerical simulation. In vitro culture experiments were carried out, and the stretch ratios of the silicone chamber were set to 0%, 5%, 10%, and 15%, respectively, to simulate the effects of different degradation stages on the growth state of endothelial cells.

**Results:**

After the stent was completely degraded, the circumferential intimal stress (strain) of the vessel was recovered to 0.137 MPa, 5.5%, which was close to the physiological parameters (0.122 MPa, 4.8%) before stent implantation. In vitro experiments showed that the endothelial cell survival rate was the highest under the condition of circumferential stress (strain) of 0.1 MPa, 5%, and all adhesion growth could be achieved.

**Conclusions:**

With the occurrence of degradation process of the stent, the circumferential stress (strain) of the intima was recovered to a range close to physiological parameters, which promotes the growth of endothelial cells. The recovery of intimal function can effectively inhibit the process of vascular restenosis. The results can provide a theoretical basis and experimental platform for the study of coronary intervention for the treatment of vascular restenosis.

## 1. Introduction

Coronary heart disease has become one of the most common and serious diseases that endanger human life. The cause of this disease is that atherosclerotic plaque formed in the coronary arteries blocks blood flow and forms a fatal long-term hidden danger. Mechanical expansion of the lesion by coronary stenting is an effective treatment to maintain blood flow. However, the usual metal stent will be permanently present in the blood vessels of the human body, causing the endothelial cells (ECs) to be damaged in the contact site, which will inevitably lead to a blood vessel remodeling reaction. Even with increasing biocompatibility of metal stents, blood vessels still produce 3~5% in-stent restenosis (ISR) [1]. Based on the stress-damage theory, the maintenance of vascular endothelial function is one of the key factors affecting the long-term efficacy of interventional therapy. After coronary stent implantation, the mechanical environment of the vascular wall changes, resulting in intimal injury, which causes blood cells to adhere and aggregate in the blood flow, and eventually causes intimal hyperplasia and restenosis [2].

Jim et al. [3] study shows that although metal stents can effectively support blood vessels to prevent their elastic retraction, long-term effects will inevitably lead to the loss of coronary blood vessel elasticity, forming a “metal jacket” phenomenon. Wykrzykowska et al. [4] analyzed the effective diastolic and systolic function of blood vessels after stent implantation, and proposed that intact endothelial function is a key factor in preventing stent thrombosis. Chiu et al. [5] used computational fluid dynamics (CFD) method and found that the wall shear stress of the vessel was abnormal after stent implantation which was closely related to intimal hyperplasia and restenosis. Therefore, the ideal stent should be matched with the physiological structure and function of the coronary artery. The implantation not only improves the local blood supply of the coronary artery, but also matches the elasticity of the blood vessel itself, thus maintaining normal endothelial function. The emerging degradable stent is based on the “vascular function recovery” theory, which can support the vessel wall in a short period of time; once the vascular remodeling was completed, it begins to degrade in the body, thereby avoiding the adverse reactions caused by the permanent implantation of the metal stent. With the dynamic degradation process of the stent, the normal diastolic and systolic function of the blood vessel can be gradually recovered, which is a significant advantage of degradable stent [6].

At present, the abnormal shear stress caused by stent implantation has been systematically and thoroughly studied [7]. Therefore, in this paper, the combination of numerical simulation and in vitro experiments was used to analyze the growth state of ECs, under the condition of dynamic degradation process of the degradable stents, and the circumferential stress (strain) of the vessel wall is constantly changing. At the same time, it provides a reference for the study of restenosis caused by coronary intervention.

## 2. Materials and Methods

### 2.1. Hyperelastic Constitutive Relation

Coronary artery is a typical soft tissue material. According to Fung's hypothesis about the quasi-elasticity of biological soft tissue, the relationship between stress and strain of arterial vessels is not single-valued during the specified loading and unloading process. They will creep under constant stress and relax under constant strain. Therefore, the material properties of the coronary arteries cannot be described by a few coefficients like homogenous materials [8]. Because the material properties show a high degree of nonlinearity, the strain potential energy (*U*) was used to express the stress–strain relationship of the coronary arteries. The constitutive equation based on the hyperelastic material proposed by Holzapfels is [9 and 10]:

(1) $$\begin{array}{c} U=\mu_{1}\left(\overset{¯}{I_{1}}-3\right)+\mu_{2}{\left(\overset{¯}{I_{1}}-3\right)}^{2}+\mu_{3}{\left(\overset{¯}{I_{1}}-3\right)}^{3}+\mu_{4}{\left(\overset{¯}{I_{1}}-3\right)}^{4}+\mu_{5}{\left(\overset{¯}{I_{1}}-3\right)}^{5}, \end{array}$$

where *μ*_1_ ~ *μ*_5_ are the material constitutive parameters, and $\overset{¯}{I_{1}}$ is the first invariant of the Cauchy–Green tensor. In order to derive a precise formula to express the mathematical relationships of hyperelastic materials, it is necessary to simplify the blood vessel into an isotropic monolayer structure and validate the acceptance level between the predicted mechanical behaviors of the material and the experimental data. In this study, the Mooney–Rivlin polynomial and Holzapfel's real blood vessel tension test data [10] were used to calculate the constitutive parameters (Table 1). At the same time, a hexahedral 8-node hybrid structural element (C3D8H) was adopted to ensure that the element would not appear excessive distortion in the dynamic analysis.

Due to the existence of residual stress (RS), the zero-load state of the blood vessel is not its zero-stress state, and the sector structure will appear when it is cut along the axis [8]. Therefore, in the case of the known opening angle (*φ* = 100°), the virtual rigid body was created in the zero-stress state of the blood vessel by the inversion method [11], so that the sheared ends were closed in the circumferential direction, and the RS is reversely loaded. Through the ABAQUS 6.14/Explicit solver (DS Simulia Inc., USA), the average value of circumferential stress (strain) of atherosclerotic blood vessels is 0.122 MPa and 4.8%, respectively, under the hemodynamic conditions of diastolic pressure of 80 mmHg and systolic pressure of 120 mmHg.

### 2.2. Construction of Finite Element Model

#### 2.2.1. Construction of Stent Degradation Model

The degradation materials of poly (lactide-co-glycolide) acid (PLGA) were selected as the research object of bioabsorbable vascular stent (BVS). Previous studies have shown that [12–14], different external conditions such as temperature, pH, degradation medium have a greater impact on its degradation behavior. However, mechanical action is a common and very important degradation factor in vivo environment. The microscopic, macroscopic structure and morphology of BVS will change significantly, which under the condition of continuous load.

The stent will produce continuous stress creep cracks during the degradation process [15], and its degradation damage was mainly manifested in the weakening ability of the material to resist deformation. Therefore, in order to realize multivariate numerical simulation by finite element method (FEM), it is necessary to establish a mass loss rate [$f\left(\overset{\sim }{\sigma },t\right)$], stress value (*σ*), and polynomial fitting equation with degradation time (*t*):

(2) $$\begin{array}{c} f\left(\overset{\sim }{\sigma },t\right)=at+bt^{2}+ct^{3}+dt^{4}+e\sigma t+f\sigma^{2}t+g\sigma^{2}t^{2}+h\sigma^{2}t^{3}. \end{array}$$

where *a*, *b* ⋯ *h* are the fitting coefficients. Since the hexahedral element of BVS exhibits random activity in different spaces, the damage rate is nonlinear. The sensitivity of each element to stress changes is expressed by the equivalent damage matrix (*C*_*d*_), so the total degradation mathematical model of BVS is:

(3) $$\begin{array}{c} F^{*}\left(\overset{\sim }{\sigma },t\right)=C_{d}^{-1}\sigma ,\;C_{d}=\left[\begin{array}{ccc} 1-c_{1}D & 0 & 0\\ 0 & 1-c_{2}D & 0\\ 0 & 0 & 1-c_{3}D \end{array}\right]. \end{array}$$

where *c*_*i*_ is consistent with the direction of the principal stress (*σ*). In the initial stage of degradation, the degradation amount (*D*) was zero, and the cross-section structure of the stent was complete. At this time, the density, elastic modulus, and Poisson ratio of stent were 1.82 g/cm^3^, 441.5 MPa, and 0.35, respectively [15]. When *D* = 1, the tensile strength of the stent material was not enough to resist the effect of the load. The cross-section structure of the stent was covered by the fracture damage and completely loses the supporting function. Through five times “mesh dependence” tests, the element type was determined to be a hexahedral 8-node reduced integration (C3D8R), and the degradation period (*T*_*f*_) was 75 weeks.

#### 2.2.2. Boundary Conditions

**Figure 1:**
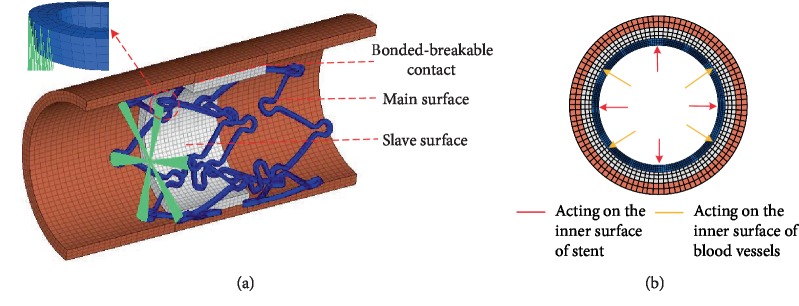
Boundary conditions (a) constraint diagram, (b) schematic diagram of load action.

Contact simulation is the core of constraint setting. The algorithm adopts ABAQUS/Explicit dynamics solution, and the constraint method uses the bonded-breakable contact. In the process of contact, the main surface node will intrude into the slave surface, and the penalty function method can be used to set the penalty coefficient to 0.2 [16], so as to limit the contact of the slave node from the main surface node within the retrieved region. In order to simulate the effects of bilateral arteries in vivo, 0.3 mm axial pre-stretching was applied to the vascular model. At the same time, in order to prevent the axial slip of the blood vessel during the expansion of the stent, it is necessary to achieve full constraint of 6 degrees of freedom on the left end node of the stent, as shown in Figure 1(a).

The load applied in the HyperMesh software (Altair Inc., USA) was mainly divided into two parts: (1) the inner surface of the stent; (2) loads are applied only to the inner surface of the vessel that is not obscured by the stent, as shown in Figure 1(b). The sinusoidal curve was used to simulate the normal stress environment of blood vessels in vivo. The cycle for 2 seconds, the diastolic pressure was 80 mmHg, and the systolic pressure was 120 mmHg. The relationship between pressure load (*y*) and time (*t*) is as follows: *y* = 20sin(*πt*−*π*/2) + 100.

### 2.3. Analysis of Vascular Mechanical Characteristics

**Figure 2:**
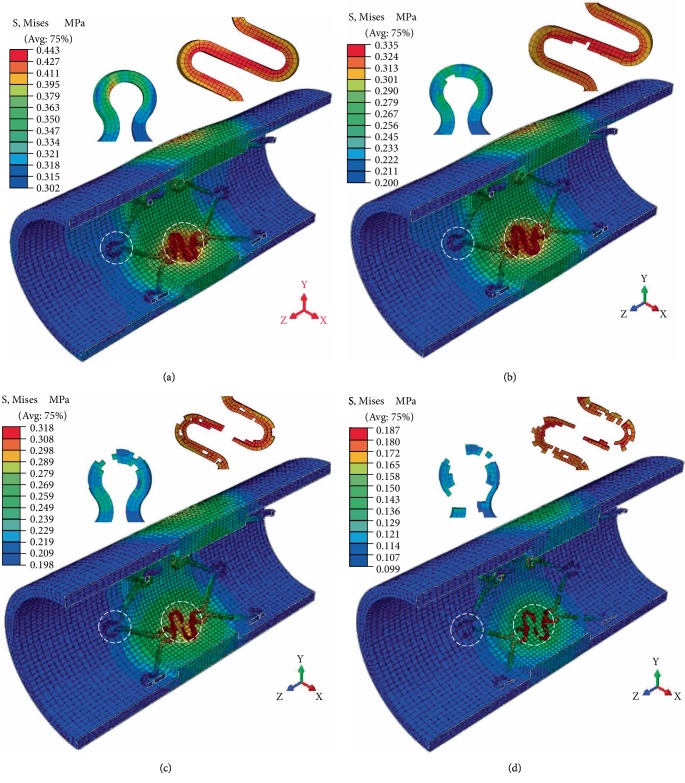
Dangerous section degradation evolution process of stent and stress distribution of blood vessels. (a) *t* = 0, (b) *t* = 0.3*T*_*f*_, (c) *t* = 0.6*T*_*f*_, and (d) *t* = 0.9*T*_*f*_.

Because of the significant stress concentration at the crowns and S-type connectors of the stent, it is considered as a dangerous section during the analysis process. The degradation variable (*D*) is used to control element deletion, and once the degradation amount is 1, the element is removed. Through numerical simulation, it was found that under the peak pressure (120 mmHg), the degradation process of BVS follows the degradation law from the surface to the inside, as shown in Figure 2.

From Figure 2, it was found that the circumferential stress of the blood vessel was not only the largest compared with the radial and axial stress values, but also has a significant effect on the functional integrity of the intimal ECs [17]. Therefore, only the circumferential stress (strain) of vascular intima was analyzed in the follow-up.

**Figure 3:**
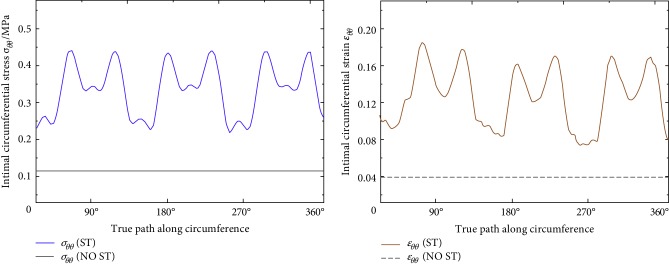
Circumferential stress and strain distribution of the intima (ST stented; NO ST stent not implanted).

Numerical simulation results show that the intimal average circumferential stress (${\overset{¯}{\sigma }}_{\theta \theta }$) in the early stage of stent implantation was 0.363 MPa, and the intimal average circumferential strain (${\overset{¯}{\varepsilon }}_{\theta \theta }$) can reach 13.1%, which was higher than the physiological value of 0.122 MPa and 4.8% of the blood vessels before stent implantation (Figure 3). After stent implantation, the vascular intima was the main stress-bearing area, so the high circumferential stress (strain) and stress concentration together, which results in the most serious mechanical damage of intimal ECs.

**Figure 4:**
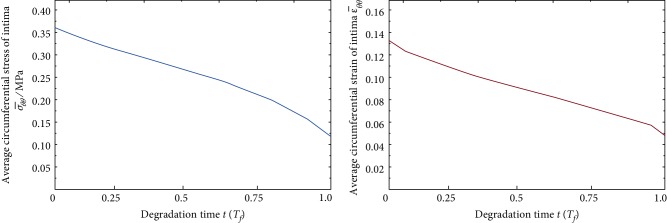
Curve of vascular circumferential stress (strain) with degradation period after implantation of BVS.

In the process of continuous degradation of BVS, the average circumferential stress (${\overset{¯}{\sigma }}_{\theta \theta }$) of the intima recovered from 0.363 MPa to 0.137 MPa, and the average circumferential strain recovered from 13.1% to 5.5% (Figure 4). Richardson et al. [18] dissected the atherosclerotic plaques of 85 patients, who died of coronary thrombosis, and pointed out that the high circumferential stress has a great correlation with the laceration of the intima. According to the experiments of Sarno et al. [19], with the degradation of BVS, the circumferential stress of the intima is gradually reduced, and the recovery of ECs makes the vascular restenosis effectively controlled.

Therefore, it can be speculated that after BVS implantation, the intima of the blood vessel is in the environment of high circumferential stress (strain), which will inhibit the growth state of ECs. With the dynamic degradation of BVS, the circumferential stress (strain) of the intima gradually recovered to the range of the physiological parameters, and this mechanical stimulation would promote the growth of ECs. At the same time, the intact endothelial function will stop the process of vascular restenosis.

## 3. In Vitro Experimental Study

In the early stage of coronary stent implantation, the circumferential stress (strain) of the intima increased sharply, and the ECs were damaged. The blood cells adhering to the intima are the main source of early stenosis in the lumen [20]. Based on the results of numerical simulation, it is predicted that with the degradation of stent, the circumferential stress (strain) of the intima gradually recovered to near physiological parameters, the growth mechanism of ECs is promoted, and the process of restenosis is effectively inhibited.

However, the mechanical transduction mechanism of circumferential stress (strain) on ECs growth is still unclear; the effects of changes in the mechanical environment of the intima and the growth state of ECs are still lacking experimental support [21]. Due to individual differences in in vivo experiments, it is still difficult to directly measure the stress on living intravascular cells [22]. Therefore, this paper uses a self-developed in vitro culture device to deeply explore how circumferential stress (strain) affects the growth state of ECs.

### 3.1. Experimental Method

**Figure 5:**
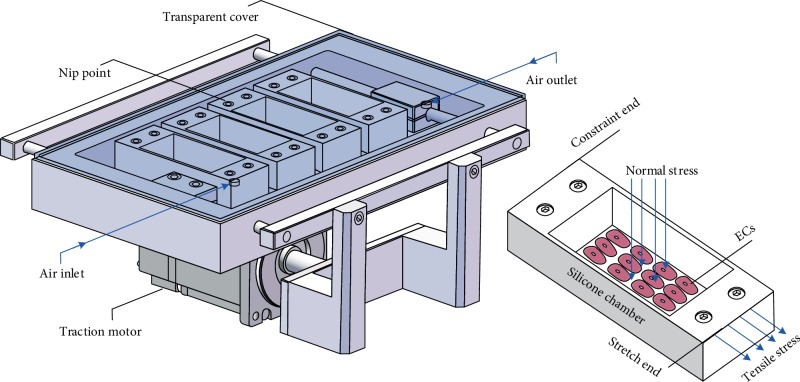
Mechanical action diagram of in vitro experimental device.

Because the circumferential stress was loaded based on rectangular substrate tensile method in vitro culture, it is also called tensile stress. The normal stress was loaded by the pressurized method in a sealed chamber, which was constant at 80~120 mmHg, and the mechanical action diagram is shown in Figure 5. In addition, due to the uniaxial tensile loading process of the silicone chamber, there is a phenomenon in which the strain distribution at the nip point is uneven. Therefore, in vitro experiment, only the uniform strain region in the middle of the chamber was studied.

Four groups of in vitro culture experiments with different stretch ratios were set, in which the static culture ratio of 0%, as a control group. In addition, in order to achieve a periodically varying tensile stress on the ECs, the dynamic culture stretch ratios were set to 5%, 10%, and 15%, respectively. The relationship between stretch ratio and time is simulated by sinusoidal curve, and the change period is 2 s.

Because the silicone chamber is linear elastic material (elastic modulus *E* is 2 MPa), there are some differences with the nonlinear constitutive characteristics of blood vessels. Therefore, the circumferential strain of the intima was 0 *T*_*f*_; 0.3 *T*_*f*_; 0.6 *T*_*f*_; and 0.9 *T*_*f*_ at the degradation period of the stent, and the stretch ratio was 15%, 10% and 5%, which have only an approximate correspondence (Table 2).

### 3.2. Experimental Results

In the experiment, the human umbilical vein endothelial cells (HUVEC) were taken in the original generation, subcultured at a ratio of 1 : 2, and 3–4 generations were used for in vitro culture experiments. After the cells were digested with 0.1% trypsin, 1 × 10^5^ cells were planted on the elastic substrate in the silicone chamber and cultured in a 37°C incubator for 24–48 h until 80%–90% of the cells meet.

**Figure 6:**
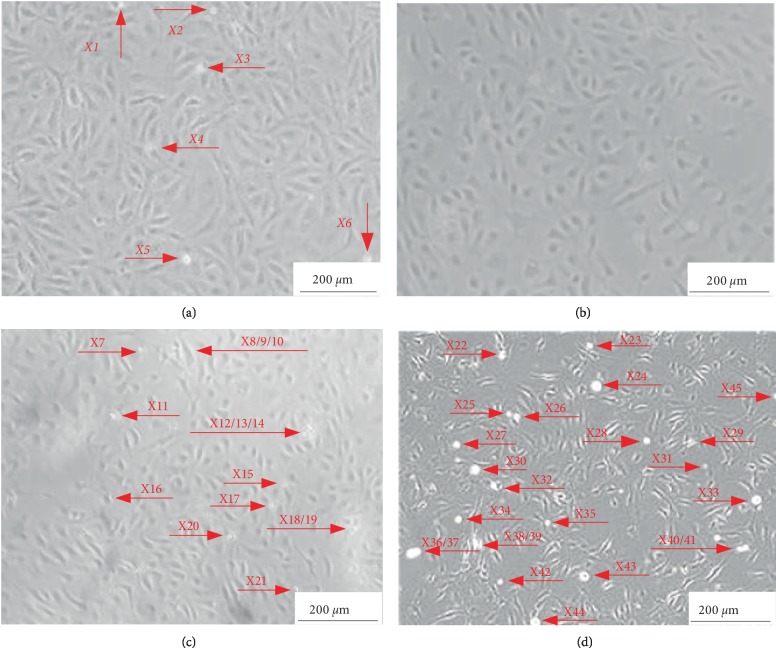
Growth state of ECs at different stretch ratios (red arrow represents cell death).

The silicone chamber of the implanted cells was placed in a 37°C, 5% CO_2_ environment, and the chamber was removed after being cultured for 12 h at four different stretch ratios (0%; 5%; 10%, and 15%). The cells were fixed with 2.5% glutaraldehyde for 10 min, and after washing with PBS, the growth state of ECs was observed under a microscope (Figure 6).

Obviously, only when the tensile stress (strain) is 0.10 MPa, 5%, which is close to the physiological parameters (*σ*_*θθ*_ = 0.122 MPa, *ε*_*θθ*_ = 4.7%), ECs grow best and can achieve all adherent growth (Figure 6(b)); since static culture has no mechanical loading and is different from the real stress environment, it is regarded as cell death after 12 h in suspension (as indicated by arrow in Figure 6(a)). However, 0.20 MPa, 10%, and 0.30 MPa, 15% belong to the super-physiological range tensile stress (strain) after stent implantation, which is different from the value of vascular tension stress (strain) before stent implantation, resulting in more cell death (Figures 6(c) and 6(d)). Moreover, hypothesis testing was performed by the jbtest function, and there was a significant positive correlation between cell death rate and abnormal high tensile stress (strain) within the required level of probability.

## 4. Conclusions and Discussions

In this paper, based on the constitutive relationship of hyperelastic and isotropic vessels, and the RS was loaded by inversion method, the finite element model of the vessel in the in vivo state was obtained. The stent-vessel coupling system model was constructed by contact simulation, and the sinusoidal curve was used to set the boundary conditions to analyze the stress distribution during the degradation process.

1. Calculate the circumferential stress (0.122 MPa) and circumferential strain (4.8%) of the vessel before coronary stent implantation. After implantation, the average circumferential stress of the intima was 0.363 MPa, and the average circumferential strain was 13.1%, which was 3.0 and 2.7 times larger than that before implantation. With the occurrence of degradation, the circumferential stress (strain) gradually recovered to 0.137 MPa, 5.5%, which is close to the physiological parameters.
2. The experimental results in vitro showed that the growth state of ECs was the best, under the condition of circumferential stress and strain (0.1 MPa, 5%) close to physiological parameters. Therefore, it is concluded that in the early stage of stent implantation, high circumferential stress (strain) of endothelium leads to obstruction of ECs repair, which is the main cause of intimal injury. Moreover, with the dynamic degradation of the stent, the circumferential stress (strain) near the physiological parameters promoted the adhesion and growth of ECs and effectively inhibited the process of vascular restenosis.

At present, interventional therapy research has expanded from a single stress to a three-dimensional stress level, from macroscopic phenomena to microscopic molecular levels. Regrettably, however, the mechanism of inducing targeted revascularization after stent implantation has not been fully elucidated [21]. Although the combination of numerical simulation and in vitro experiments is used to illustrate the clinical advantages of degradable stents, there are still several issues to be further studied:

1. Due to individual differences, the degradation rate of BVS is difficult to control. Incompletely degraded stents are prone to block distal blood vessels, causing angina or myocardial infarction, which severely limits the application and development of degradable stents. Therefore, it is necessary to establish a more accurate mathematical model of degradation to predict the appropriate degradation time and degradation curve [23].
2. The properties of plaque in atherosclerotic blood vessels are very complicated, which is one of the research hotspots in recent years. Due to the limitations of current medical imaging techniques and measurement methods, the correlation between mechanical factors and plaque formation has not been fully determined. Moreover, with the change of plaque morphology, the mechanical characteristics of the whole blood vessel will also change. Therefore, in numerical simulation, it is necessary to consider the influence of plaque property changes on vascular constitutive relations [24].
3. In this paper, it is preliminarily determined by in vitro experiments that the ECs have the highest survival rate under the conditions of circumferential stress (*σ*_*θθ*_ = 0.1MPa and circumferential strain (*ε*_*θθ*_ = 5%), and the precise mechanical parameters need to be further explored in subsequent experiments. Moreover, the growth inhibition of ECs by high circumferential stress (strain) is only one of the mechanical factors. After coronary stent implantation, the stress concentration and flow shear effect will cause the ECs repair to be hindered. Therefore, in subsequent in vitro experiments, the influence of other mechanical factors on the growth state of ECs needs to be considered [25].

## Figures and Tables

**Table 1:** Vascular wall and plaque constitutive parameters (MPa).

Material model	Constitutive parameters					
Polynomial coefficients	*μ*_1_	*μ*_2_	*μ*_3_	*μ*_4_	*μ*_5_	Compressibility (*D*_1_)
Blood vessel	0.00652	0.0489	0.00926	0.76	−0.043	0
Plaque	0.04	0.03	0.02796	—	—	0

**Table 2:** Correspondence between degradation period and stretch ratio ($\overset{¯}{x}\pm s,n=5$).

Degradation period *t*/*T*_*f*_	Average circumferential stress of intima ${\overset{¯}{\sigma }}_{\theta \theta }$/MPa	Average circumferential strain of intima ${\overset{¯}{\varepsilon }}_{\theta \theta }$/%	Stretch ratio/%
0	0.363 ± 0.004	13.1 ± 0.213	15
0.3	0.304 ± 0.003	11.7 ± 0.141	10
0.6	0.247 ± 0.002	9.6 ± 0.075	
0.9	0.137 ± 0.002	5.5 ± 0.061	5

## Data Availability

The data used to support the findings of this study are available from the corresponding author upon request.
